# Supplementary material for: Acceptability of smart locker technology for dispensing chronic disease medication among patients and healthcare providers in Nigeria
Source: PLoS One. 2024 Mar 7;19(3):e0294936. doi: 10.1371/journal.pone.0294936 (PMC10919599; doi:10.1371/journal.pone.0294936)
Supplement: S1 Appendix — (DOCX) [file pone.0294936.s001.docx]

**Appendix 1.** **Distribution of States and Facilities**

| State | Facility |
| --- | --- |
| Adamawa | Michika General Hospital |
|  | Mubi General Hospital |
|  | Numan General Hospital |
|  | Song Cottage Hospital |
|  |  |
| Akwa Ibom | Ikpe Ikot Nkon General Hospital |
|  | Etim Ekpo General Hospital |
|  | Ibiono Handmaids Hospital |
|  | Ikono General Hospital |
|  | Ikot Ekpene General Hospital |
|  | Ikot Ekpene Primary Health Centre |
|  | West Itam Primary Health Center |
|  | Mbiabong Itam Health Center |
|  | Nto Edino Comprehensive Health Centre |
|  | Ukana Cottage Hospital |
|  | Ukpom Abak General Hospital |
|  | Urua Inyang Primary Health Centre |
|  |  |
| Benue | Aliade General Hospital |
|  | St. Gregory Health Center |
|  | St. Vincents Hospital |
|  | Adikpo General Hospital |
|  | Nongu u Kristu ke Sudan hen Tiv (NKST) Hospital - Jato Aka |
|  | St. Monica's Hospital - Adikpo |
|  | St. Mary's Hospital - Okpoga |
|  | Oturkpo General Hospital |
|  | Nongu u Kristu ke Sudan hen Tiv (NKST) Hospital - Mbaakon |
|  | Sev-Av Foundation - Vandeikya |
|  | St. Thomas Hospital - Ihugh |
|  | Vandeikya General Hospital |
|  |  |
| Cross river | Obanliku General Hospital |
|  | Ogoja Catholic Maternity Hospital |
|  | Ogoja General Hospital |
|  | Sacred Heart Catholic Hospital |
|  |  |
| Niger | General Hospital -Bida |
|  | Federal Medical Centre - Bida |
|  | General Hospital Mokwa |
|  | General Hospital Lapai |
|  | General Hospital New Bussa |
|  | General Hospital Kontagora |
|  |  |
