# Supplementary material for: Acceptability of smart locker technology for dispensing chronic disease medication among patients and healthcare providers in Nigeria
Source: PLoS One. 2024 Mar 7;19(3):e0294936. doi: 10.1371/journal.pone.0294936 (PMC10919599; doi:10.1371/journal.pone.0294936)
Supplement: S3 Appendix — (PDF) [file pone.0294936.s003.pdf]

FEASIBILITY AND ACCEPTABILITY OF “SMART LOCKERS” FOR DISPENSING CHRONIC DISEASE MEDICATION IN NIGERIA

PROTOCOL

GEORGETOWN UNIVERSITY

Center for Global Health Practice and Impact

AUGUST 23, 2021

GEORGETOWN UNIVERSITY – CENTER FOR GLOBAL HEALTH PRACTICE AND IMPACT
No 2, Babatope Ajakaiye Crescent, Jahi District, Abuja

Table of Contents

Participating Institutions and Roles iii

Investigators and Roles iv

PROTOCOL SUMMARY vi

INTRODUCTION 7

Background Information 7

Problem statement 7

Justification 8

Aim and Objectives 8

METHODS 8

Study design 8

Study population 8

Sample Size Determination 9

Sampling Technique 9

Data sources/Collection Methods 10

Patients' survey 10

Healthcare workers' survey 11

Data Management 11

Data entry 11

Data Quality Assurance 12

Data Analysis 12

ETHICAL CONSIDERATIONS 13

Limitations 13

REFERENCES 13

ANNEXES 16

Annex 1: Healthcare Provider Survey Questionnaire 16

Annex 2: Patient Survey Questionnaire 20

Participating Institutions and Roles

Institution	Location	Role
Georgetown University – Center for Global Health Practice and Impact	Abuja (Nigeria) and Washington DC (USA)	• Develop protocol<br>• Coordinate and conduct data collection<br>• Analyze results<br>• Interpretation of results for dissemination<br>• Manuscript development<br>• Publication management

Contact:

Dr. Ibrahim Bola Gobir
Field Resident Director
Georgetown University – Centre for Global Health Practice and Impact
Telephone: +234 8023265259
Email: ibg7@georgetown.edu

Investigators and Roles

Name	Title/affiliation	Role on project
Dr. Ibrahim Bola Gobir	Assistant Clinical Professor of Medicine & Field Resident Director<br><br>Georgetown University – Centre for Global Health Practice and Impact	Principal Investigator (PI):<br>• Overall direction and management of the project<br>• Protocol development<br>• Coordinate ethical approval<br>• Oversight of project implementation<br>• Interpretation of results for dissemination<br>• Manuscript development<br>• Publication management
Dr. Ayodotun Olutola	Assistant Clinical Professor of Medicine & Co-Resident Field Director<br><br>Georgetown University – Centre for Global Health Practice and Impact	Co-PI:<br>• Overall direction and management of the project<br>• Provide input on protocol development<br>• Coordinate ethical approval<br>• Oversight of project implementation<br>• Interpretation of results for dissemination<br>• Manuscript development
Piring'ar Mercy Niyang	Director, Health Program<br><br>Georgetown University – Centre for Global Health Practice and Impact	Co-PI:<br>• Overall direction and management of the project<br>• Provide input on protocol development<br>• Coordinate ethical approval<br>• Oversight of project implementation<br>• Interpretation of results for dissemination<br>• Manuscript development

Havilah Onyinyechi Nnadozie	Research assistant,<br><br>Georgetown University – Centre for Global Health Practice and Impact	Research assistant:<br><br>● Provide input on protocol development<br>● Manuscript development<br>● Survey application transcription<br>● Data management
Dr. Samson Agboola	Statistician,<br><br>Georgetown University – Centre for Global Health Practice and Impact	Research Statistician:<br><br>● Provide input on protocol development<br>● Monitor adherence to protocol<br>● Data analysis<br>● Manuscript development<br>● Publication management

PROTOCOL SUMMARY

**Title**	Feasibility and acceptability of “smart lockers” for dispensing chronic disease medications in Nigeria
**Background**	A smart locker is an automated delivery machine that has compartments used for the storage of items. The user can have access to the stored item by typing in an electronic access code that is generated once an item is deposited into the storage unit. In South Africa, where the smart lockers are being used to dispense drugs to persons living with chronic diseases, has resulted in the enhancement of patient-centered healthcare services, reduced strain on health resources and improved patient health outcomes.
**Purpose**	The aim of this study is to assess the feasibility and acceptability of smart lockers for dispensing chronic disease medications in Nigeria
**Objectives**	1. To describe the barriers to accessing and providing care for chronic illnesses among patients and healthcare providers in Nigeria<br>2. To assess acceptability of smart lockers for drug refills by persons living with chronic illnesses<br>3. To assess the acceptability of smart lockers for dispensing drug refills by healthcare providers for people living with chronic illnesses<br>4. To describe the patient and healthcare providers preferences for the use of the smart lockers for dispensing chronic disease medications in Nigeria
**Study Design**	Descriptive cross-sectional study
**Study population**	1. Health workers who provide healthcare services to individuals living with chronic diseases<br>2. Persons living with one or more of the following chronic illnesses: HIV, diabetes, hypertension, stroke, chronic kidney disease, cancer, Tuberculosis, etc.<br>3. Caregivers of people living with chronic illnesses (children and the elderly)
**Study Duration**	Four weeks
**Approach**	384 healthcare providers and 601 patients who meet the inclusion criteria to participate in the study will be recruited through United State Government President Emergency Plans for AIDS Relief (PEPFAR) program Implementing partners (IPs) across the sites they support in Nigeria and through distribution on social networks such as private messaging, electronic mails, social media platforms such as WhatsApp, LinkedIn, Instagram and Facebook. Data collection for the survey will be conducted using both paper-based forms among PEPFAR program IPs and an online survey on RedCap.

INTRODUCTION

Background Information

In Nigeria, chronic diseases such as cancer, diabetes, cardiovascular disease and chronic respiratory disease account for 29% of all deaths and are projected to become the leading cause of morbidity and mortality by 2030. (WHO, 2018). Chronic disease management usually follows irregular and sporadic patterns putting patients at risk of developing long-term complications and death. (Mayston, Ebhohimen and Jacob, 2020). This is different from the treatment paradigms adopted in HIV programs, whereby people living with HIV (PLHIV) have access to cross-cutting care models that focuses on continuity and retention, routine monitoring and promotion of healthy lifestyles necessary to achieve better health outcomes (Duffy *et al.*, 2019).These differentiated service delivery (DSD) models seek to achieve patient-centered care and ensure that treatment goals for PLHIV are achieved. (UNAIDS, 2015; Adjetey, Obiri-yeboah and Dornoo, 2019). Patient centered service delivery models such as community-based drug refills, peer and advocacy groups, multi-month drug scripting and dispensing and smart lockers for dispensing drug refills have emerged particularly in the treatment of PLHIV to address these challenges encountered in delivering care to patients. (Abelman *et al.*, 2020; Adjetey, Obiri-yeboah and Dornoo, 2019; Eshun-wilson *et al.*, 2019; Long *et al.*, 2020; Magadzire, Marchal and Ward, 2016). A smart locker is an automated delivery machine that has compartments used for the storage of items. A unique key or electronic code is generated each time a parcel is delivered to the storage unit. The user will have access to the parcel by typing in the electronic code. (Refaningati, Nahry, Tankdung and Kusuma, 2020). Across the world, they have been used for fast, easy and convenient product delivery in shipping, banking and healthcare. (Carameli, Eisenman, Blevins, d'Angona and Glik, 2013; Ehrenkranz, Grimsrud, Holmes, Preko, and Rabkin, 2021).

The promotion of patient-centered treatment services has been acknowledged as an essential step in achieving global development. In 2019, the UN also published a political commitment to achieving Universal Health Coverage (UHC) which envisions access to quality healthcare by all. (WHO, 2019). This can be achieved by the adoption of truly patient-centered treatment paradigms that address the chronic disease epidemic.

Problem statement

The chronic disease burden in Nigeria is high and is only projected to increase with time. Challenges encountered in service delivery include strain on the health workforce and healthcare facility resources, long waiting times when persons visit healthcare facility, transportation costs to access care, and increased risk of transmission of nosocomial infections. Furthermore, the COVID-19 pandemic has led to revamped emphasis on the need to strengthen and promote sustainable patient-centered care that is not limited to the confines of healthcare facilities. (Faronbi, Ademuyiwa and Olaogun, 2020; Johns and Baruwa, 2016; Lujintanon *et al.*, 2021; WHO, 2020).

Justification

The use of smart lockers to dispense drug refills for persons living with chronic diseases has led to reduced patient waiting times, decongestion of healthcare facility and resources, creation of employment opportunities for more health workers and service delivery that takes the patients' comfort and convenience into account. A truly patient-centered service delivery model. (Duncombe *et al.*, 2015; Bygrave, Golob, Wilkinson, Roberts and Grimsrud, 2020). However, no research has been conducted on the feasibility of the smart lockers for dispensing chronic disease medications including ART in Nigeria.

Aim and Objectives

The aim of this study is to assess the feasibility and acceptability of smart lockers for dispensing chronic disease medications in Nigeria

The objectives of the study are:

1. To describe the barriers to accessing and providing care for chronic illnesses among patients and healthcare providers in Nigeria
2. To assess acceptability of smart lockers for drug refills by persons living with chronic illnesses
3. To assess the acceptability of smart lockers for dispensing drug refills by healthcare providers for people living with chronic illnesses
4. To describe the patients' and healthcare providers' preferences for the use of smart lockers in dispensing chronic disease medications in Nigeria

METHODS

Study design

This study will use a descriptive cross-sectional study design. Primary data will be collected in the form of participants' responses to questions in order to determine their perceptions toward the use of smart lockers for dispensing chronic disease medications in Nigeria.

Study population

The target populations for this study are

1. Health workers who provide healthcare services to individuals living with chronic diseases
2. Persons living with one or more of the following chronic illnesses: HIV, diabetes, hypertension, stroke, chronic kidney disease, Cancer, TB, etc.
3. Caregivers of persons living with chronic illnesses (children and the elderly)

Inclusion criteria

1. Persons that require regular drug refills for their chronic illness
2. Healthcare providers for patients with chronic illness

Sample Size Determination

Patient/ caregivers of persons living with chronic illnesses

According to Faronbi, Ademuyiwa and Olaogun (2020) the percentage of chronic diseases in Nigeria is about 64.9% of the population. This was used as a proxy to estimate the population prevalence of chronic diseases given that the population of Nigeria is 200 million. (United Nations Population Fund, 2021). This was entered into Raosoft® sample size calculator at 4% margin of error, 95% confidence interval and 50% response distribution to yield a minimum sample size of 601. The population of caregivers is assumed to be proportional to that of persons living with chronic illnesses and as such is not calculated separately.

Healthcare workers (HCW)

According to Adebayo, Labiran, Emerenini and Omoruyi (2016), the estimated number of HCW was 621,205. This was used as a proxy to HCW that provide care to patients with chronic illness. This was inputted into Raosoft® sample size calculator at 5% margin of error, 95% confidence interval and 50% response distribution to yield a minimum sample size of 384.

Sampling Technique

Mixed methods will be used to recruit participants for the study.

Recruitment route 1: ART programs' implementing partners in Nigeria.

Patients with chronic illnesses and HCW will be identified through ART programs' IP; CCCRN, CCFN, JHPIEGO and APIN, within their sites in Nigeria. Copies of the survey will be printed and distributed among each of the IPs. Stratified random sampling will be used by each of the IPs to identify 120 patients living with chronic illness (or caregivers of children and elderly persons living with chronic illnesses) and 80 HCW that meet the inclusion criteria from the sites they manage in Nigeria. The participants will be stratified on the basis of gender i.e., 50% male and 50% female, to ensure adequate coverage of the target population. Those identified will be asked to participate in the study and will be provided with a description of the study and study objectives. Those who agree to participate in the study will be provided with the survey questionnaire to complete. The IPs or their representatives within each study site will serve as field supervisors for data collection during the study.

Recruitment route 2: Advertising online including social media

The survey will be advertised online via social networks including but not limited to private messaging, electronic mails, social network sites and groups through platforms such as WhatsApp, Facebook, Instagram and LinkedIn. Potential participants will be privy to information about the study and screened based on the inclusion criteria for the study.

Data sources/Collection Methods

Patients' survey

A semi-structured questionnaire has been developed for the patient survey. The questionnaire consists of 37 questions. The questions include information on socio-demographic characteristics, knowledge of smart lockers, current barriers to accessing treatment, perception of using the smart lockers for the collection of medication, potential benefits and challenges with using the smart lockers for collecting medication. The survey will be administered in English language and will be pre-tested for accuracy, acceptability and comprehensibility before the study is commenced. No personally identifiable information will be collected from the participants. All data will be confidentially treated. There are no adverse effects on the rights and welfare of participants. Data collection will be conducted for 4 weeks.

Paper-based data collection

Within the ART and outpatient clinics of the facilities where HIV program IPs operate, attending physicians and nurses will be given copies of the patient questionnaires by the field supervisors representing the IPs. These will identify potential participants who meet the inclusion criteria from among patients visiting the facility and/or their caregivers and invite them to participate in the study. They will explain that (1) the patients (or their caregiver) must read the research information (2) consent is voluntary and (3) they should tick the agreement checkbox on the questionnaire form if they are able and willing to participate in the survey. It is considered appropriate that informed consent has been obtained once a potential participant ticks the agreement checkbox. Each patient will complete the questionnaire independently and return the form to the physician upon completion. It will take about 10 minutes to complete the questionnaire. All completed forms will be transferred from the physician or HCW to the field supervisor, who will then forward it to the PI at the Georgetown University Nigeria office. The forms will be placed in a sealed envelope during transit, to ensure integrity of the data collected.

Online survey

The study will be deployed as an online survey to be distributed via social networks in all media handles across Nigeria within a two-week period. These media will include but not limited to private messaging, electronic mails, social networking sites and groups through platforms such as WhatsApp, Facebook, Instagram and LinkedIn. The survey questionnaire will be transcribed into a survey platform; RedCap with validation checks to curtail the incidence of missing data. A participant will be directed to the entry page of the survey by clicking on a hyperlink on their mobile devices or computers. A click on the submit button marks the end of the survey. There will be no room to retrieve forms for editing after submission. Each entry into the survey will be assigned a unique ID consisting of the time and date stamp at which the response was submitted.

Healthcare workers’ survey

A semi-structured questionnaire has been developed for the HCW survey. The questionnaire consists of 37 questions. The questions include socio-demographic characteristics, knowledge of smart lockers, current barriers to providing treatment to patients living with chronic illnesses, perception of using the smart lockers to dispense chronic disease medication, potential benefits and challenges with using the smart lockers for dispensing chronic disease medication. The survey will be administered in English language and will be pre-tested for accuracy, acceptability and comprehensibility before the study is commenced. No personally identifiable information will be collected from the participants. All data will be confidentially treated. There are no adverse effects on the rights and welfare of participants. Data collection will be conducted for 4 weeks.

Paper-based data collection

Representatives of the IPs will serve as field supervisors for data collection. They will be required to identify potential participants for the survey. They will then distribute the HCW survey forms to HCW that meet the inclusion criteria attending the ART and outpatient clinics in the facility. The field supervisor will be required to explain the following (1) that the participant must read the accompanying research information (2) that consent is voluntary and (3) that they should tick the agreement checkbox in the questionnaire form if they are willing and able to participate in the survey. It is considered appropriate that informed consent has been obtained once a participant ticks the agreement checkbox. Each participant will complete the questionnaire independently and return it to the field supervisor for the facility. It will take about 10 minutes to complete the questionnaire. All completed forms will be collated by the field supervisor and transferred to the PI at the Georgetown University Nigeria office. The forms will be placed in a sealed envelope during transit, to ensure integrity of the data collected.

Online survey

The study will be deployed as an online survey to be distributed via social networks in all media handles across Nigeria within a two-week period. These media will include but not limited to private messaging, electronic mails, social networking sites and groups through platforms such as WhatsApp, Facebook, Instagram and LinkedIn. The survey questionnaire will be transcribed into a survey platform; RedCap with validation checks to curtail the incidence of missing data. A participant will be directed to the entry page of the survey by clicking on a hyperlink on their mobile devices or computers. A click on the submit button marks the end of the survey. There will be no room to retrieve forms for editing after submission. Each entry into the survey will be assigned a unique ID consisting of the time and date stamp at which the response was submitted.

Data Management

Data entry

The online survey will be accessed using minimal data connectivity from multiple network options on the participant’s device. All survey forms completed from the paper-based

survey will be retrieved from the participants by the field supervisors and returned to the PI. The responses from each form will then be transcribed into the online survey platform, Redcap by the research assistant, for data collation and analysis. Each entry will be assigned a unique ID consisting of the time and date stamp at which the response was submitted, to enable traceability of entries and to ensure data consistency and validity.

The submitted responses will be synchronized and uploaded directly into a central database hosted in a server in Georgetown university office in Nigeria if there is connectivity. If not, it will be saved temporarily on a device and then synced once a secure connection is established. Data will be protected during transmission using encryptions as well as protected by a firewall to manage access with secure authentication on both the server machine and the database itself.

Data Quality Assurance

To improve data quality, the online survey and database will include validation checks for missing fields and invalid responses. Data will be cleaned to identify and remedy invalid responses and ensure the standardization of variable responses. Each submitted response will be assigned a unique identification code consisting of the time and date stamp at which the response was submitted. There will be a regulation on adherence to study protocol, data completeness and accuracy.

Measurement of Variables

The categorical variables of the study will include gender, age, marital status, state of residence, educational attainment and employment status. The outcome variables differ according to the type of participant.

Healthcare workers

1. Perception on using the smart lockers to dispense drugs to patients with chronic illnesses
2. Factors associated with the use of smart lockers for dispensing drugs to patients with chronic illnesses
3. Benefits and challenges associated with the use of smart lockers for dispensing chronic disease medications

Patients and caregivers of persons living with chronic illnesses

1. Barriers to accessing treatment for chronic diseases
2. Perception on using the smart lockers for collecting prescribed medication
3. Factors associated with the use of smart lockers for collecting medication
4. Benefits and challenges associated with the use of smart lockers for collecting chronic disease medication

Data Analysis

Data will be analyzed using STATA version 15. Data analysis will involve descriptive statistics, Chi square, Analysis of variance and correlation statistics. The 95% confidence

intervals (CI) and p-values obtained will be estimated in two tailed form and statistical difference of associations will be determined at an alpha level of 0.05.

ETHICAL CONSIDERATIONS

Informed consent

The entry page of both the paper based and online surveys will contain information on the study objectives, eligibility criteria, data privacy and researchers' disclaimers. Informed consent will be sought from participants. A click on the "I agree" button on the preview page of the online survey is sufficient to provide consent. Likewise, if a participant ticks the "I agree" checkbox on the paper-based survey, it is considered sufficient to provide informed consent. Entries from participants who do not meet the inclusion criteria will not be processed for data analysis.

Confidentiality

All entries will be recorded anonymously. Personally identifiable information will not be collected from the participants. Privacy of the subjects' information will be guaranteed.

Risks and benefits

There are no adverse effects that will affect the right and welfare of the subjects. There are no direct benefits associated with participation in the study.

Ethical clearance

The study protocol will be submitted for review and approval to the Nigeria Health Research Ethics Committee (NHREC). Permission will also be sought from the administrators of the health facilities where data collection will be conducted.

Limitations

There is likely to be a degree of self-selection bias. For example, the decision to participate in the study may reflect some inherent bias in the characteristics/traits of the participants towards the use of online surveys. In addition, a limitation of online surveys is the inability to verify responses are from the target population. This can either lead to the sample not being representative of the population being studied or exaggerating some findings from the study. Study objectives, eligibility criteria and informed consent will be elaborate to mitigate the impact of these limitations on the study.

REFERENCES

1. Abelman, R., Alons, C., Stockman, J., Teri, I., Grimsrud, A., Ombija, M., Makwindi, C., Odionyi, J., Tumbare, E., Longwe, B., Bonou, M., Songoro J., Mugumya, L. and Cohn, J. (2020). Implementation of differentiated service delivery for paediatric HIV care and treatment: opportunities, challenges and experience from seven sub- Saharan African countries. *Family Medicine and Community Health.* 8, e000393. doi: 10.1136/fmch-2020-000393.

2. Adebayo, O., Labiran, A., Emerenini, F. C. and Omoruyi, L. (2016). Health Workforce for 2016-2030: Will Nigeria have enough? *International Journal of Innovative Healthcare Research.* 4(1), 9-16.
3. Adjetey, V., Obiri-yeboah, D. and Dornoo, B. (2019). Differentiated service delivery: a qualitative study of people living with HIV and accessing care in a tertiary facility in Ghana. *BMC Health Services Research.* 19,95.
4. Bygrave, H., Golob, L., Wilkinson, L., Roberts, T. and Grimsrud, A. (2020). Let's talk chronic disease: can differentiated service delivery address the syndemics of HIV, hypertension and diabetes? *Current Opinion.* 15(4), 256–260. doi: 10.1097/COH.0000000000000629.
5. Carameli, K. A., Eisenman, D., Blevins, J., d'Angona, B. and Glik, C. D. (2013). Planning for chronic disease medications in disaster: perspectives from patients, physicians, pharmacists, and insurers. *Disaster Medicine and Public Health Preparedness*. 7(3), 257–265. doi: 10.1001/dmp.2010.46.
6. Duffy, M., Sharer, M., Davis, N., Eagan, S., Haruzivishe, C., Katana, M., Makina, N. and Amanyeiwe, U. (2019). Differentiated Antiretroviral Therapy Distribution Models: Enablers and Barriers to Universal HIV Treatment in South Africa, Uganda, and Zimbabwe. *Journal of the Association of Nurses in AIDS Care*. 30(5), E132–E143. doi: 10.1097/JNC.0000000000000097.
7. Duncombe, C., Rosenblum, S. Hellmann, N., Holmes, C., Wilkinson, L., Biot, M., Bygrave, H., Hoos, D. and Garnett, G. (2015). Reframing HIV care: Putting people at the centre of antiretroviral delivery. *Tropical Medicine and International Health*. 20(4), 430–447. doi: 10.1111/tmi.12460.
8. Ehrenkranz, P., Grimsrud, A., Holmes, C., Preko, P. and Rabkin, M. (2021). Expanding the Vision for Differentiated Service Delivery: A Call for More Inclusive and Truly Patient-Centered Care for People Living with HIV. *Journal of Acquired Immune Deficiency Syndrome*. 86(2), 147–152.
9. Eshun-wilson, I., Mukumbwa-Mwenechanya, M., Kim, H., Zannolini, A., Mwamba, P. C., Dowdy, D., Kalunkumya, E., Lumpa, M., Beres, K. L., Roy, M., Sharma, A., Topp, M. S., Glidden, V. D., Padian, N., Ehrenkranz, P., Sikazwe, I., Holmes, B. C., Bolton-Moore, C. and Geng, H. E. (2019). Differentiated Care Preferences of Stable Patients on Antiretroviral Therapy in Zambia: A Discrete Choice Experiment. 81(5), 540–546.
10. Faronbi, J. O., Ademuyiwa, I. Y. and Olaogun, A. A. (2020). Patterns of chronic illness among older patients attending a university hospital in Nigeria. *Ghana Medical Journal.* 54(1), 42–47. doi: 10.4314/gmj.v54i1.7.
11. Johns, B. and Baruwa, E. (2016). The effects of decentralizing anti-retroviral services in Nigeria on costs and service utilization: Two case studies. *Health Policy and Planning*. 31(2), 182–191. doi: 10.1093/heapol/czv040.
12. Long, L., Kuchukhidze, S., Pascoe, S., Nichols, B., Fox, M., Cele, R., Govathson, C., Huber, C., Flynn, D. and Rosen, S. (2020). Retention in care and viral suppression in differentiated service delivery models for HIV treatment delivery in sub-Saharan Africa: a rapid systematic review. *Journal of the International AIDS Society.* 23(11), e25640. doi: 10.1002/jia2.25640.

13. Lujintanon, S., Amatavete, S., Sungship, T., Seekaew, P., Peelay, J., Mingkwanrungruang, P., Chinbunchorn, T., Teeratakulpisarn, S., Methajittiphan, P., Leenasirima, P., Norchaiwong, A., Nilmanat, A., Phanuphak, P. Ramautarsing, A. R. and Phanuphak, N. (2021). Client and provider preferences for HIV care: Implications for implementing differentiated service delivery in Thailand. *Journal of the International AIDS society.* 24, e25693. doi: 10.1002/jia2.25693.
14. Magadzire, B. P., Marchal, B. and Ward, K. (2016) "Novel models to improve access to medicines for chronic diseases in South Africa: An analysis of stakeholder perspectives on community-based distribution models," *Journal of Pharmaceutical Policy and Practice*, 9(1), 28. doi: 10.1186/s40545-016-0082-6.
15. Mayston, R., Ebhohimen, K. and Jacob, K. (2020). Measuring what matters-information systems for management of chronic disease in primary healthcare settings in low and middle-income countries: Challenges and opportunities. *Epidemiology and Psychiatric Sciences*. 29, e127. doi: 10.1017/S204579602000030X.
16. Refaningati, T., Nahry, Tankdung, W. S. E. and Kusuma, A. (2020). Analysis of characteristics and efficiency of smart locker system (Case study: Jabodetabek). *Evergreen*, 7(1), 111–117. doi: 10.5109/2740966.
17. UNAIDS .(2015). Understanding Fast-Track Targets. Accelerating action to end the AIDS epidemic by 2030. Retrieved 8 March, 2021 from https://www.unaids.org/sites/default/files/media_asset/201506_JC2743_Understanding_FastTrack_en.pdf
18. United Nations Population Fund (2021). World Population dashboard Nigeria. Retrieved 30 June, 2021 from https://www.unfpa.org/data/world-population/NG
19. World Health Organization (2018). Noncommunicable Diseases Country profiles 2018. World Health Organization. Retrieved 29 June 2021 from https://apps.who.int/iris/handle/10665/274512
20. World Health Organization (2019). WHO HIV Policy Adoption and Implementation Status in Countries. Retrieved 18 June, 2021 from https://apps.who.int/iris/rest/bitstreams/1327403/retrieve
21. World Health Organization (2020). Strengthening NCD Service Delivery through UHC Benefit Package: technical meeting report, Geneva, Switzerland, 14-15 July, 2020. Geneva: World Health Organization. https://apps.who.int/iris/rest/bitstreams/1327403/retrieve

ANNEXES

Annex 1: Healthcare Provider Survey Questionnaire

HEALTH CARE PROVIDER SURVEY ON THE FEASIBILITY OF SMART LOCKERS FOR DISPENSING CHRONIC DISEASE MEDICATION IN NIGERIA

This survey contains questions that are designed to gather your perception on: the barriers to providing care to patients with chronic illness, the use of smart lockers for dispensing chronic disease medication, challenges and benefits of using the smart lockers for dispensing chronic disease medication. The goal of the survey is to assess the feasibility and acceptability of using smart lockers for dispensing chronic disease medication in Nigeria. Your participation in this study is voluntary. No personal information will be collected from you. Your response will be treated confidentially. If you are willing and able to participate in this study, kindly tick the box below.

☐ I agree to participate in this study

1. Gender

   Male ☐ Female ☐

2. Age (as at last birthday)

☐ 18-35 years ☐ 36-60 years ☐ Above 60 years

3. State of residence ___________________________ LGA of residence _________________________

4. Marital status

☐ Single ☐ Married ☐ Separated ☐ Divorced ☐ Widowed

5. What is your highest educational qualification?

☐ No formal education ☐ First school leaving certificate ☐ WAEC/GCE/NECO

☐ Bachelor's Degree/HND ☐ Masters/MBBS ☐ PhD/DrPH/DA/DBA

Others____ (specify)

6. What is your primary role as a healthcare provider?

☐ Doctor ☐ Nurse ☐ Pharmacist ☐ Laboratory scientist

☐ Behavioural therapist ☐ Physiotherapist ☐ Nutritionist/Dietician

☐ M&E officer ☐ HIV Case manager ☐ HIV Counsellor ☐ Community Health Extension worker

☐ Other ______________________ specify

7. What is the name of the facility where you work as a healthcare provider?

____

8. How long have you served in your current role?

☐ Less than 6 months ☐ 6 months-1 year ☐ 1 – 5 years ☐ More than 5 years

9. In your current role, do you provide specialized healthcare services to people living with chronic diseases?
   ☐ Yes ☐ No
10. Which chronic illnesses do you provide specialized services for? (select all that apply)

☐ HIV ☐ Diabetes ☐ Cancer ☐ Hypertension ☐ Stroke ☐ Chronic kidney disease

☐ Tuberculosis ☐ Other ____ (specify)

11. Which of these services do you provide to patients with chronic illnesses?

☐ Medical consultation ☐ Diagnostic services ☐ Dispensing drugs

☐ Behavioural therapy ☐ Physical therapy ☐ Patient documentation

☐ Others ____ (specify)

12. On an average, how many patients with chronic illness do you attend to weekly?

☐ Less than 10 patients ☐ 10 – 50 patients ☐ 50 – 100 patients ☐ More than 100 patients

13. Which of these is a challenge you have faced in delivering services to patients?

☐ Long working hours ☐ High volume of patients ☐ Physical fatigue ☐ Emotional fatigue
☐ Competing work responsibilities ☐ Limited resources in the healthcare facility

14. In which of these instances have you used a locker for storage of items? (select all that apply)

☐ Banking hall ☐ Post office box ☐ Gym locker ☐ Spa or beauty salon ☐ Supermarket
☐ I have not used a locker before (skip to Section 2)
☐ Other _________________________________ (specify)

15. Which of these features does the locker you have used possess?

☐ Storage compartment ☐ Padlock and key ☐ Electronic keypad ☐ Camera
☐ Display screen ☐ Audio communication system

16. Have you made use of a storage locker that has these features: Storage compartment, Electronic keypad, Camera, Audio communication system

☐ Yes ☐ No

17. How have you made use of the locker?

☐ Posting of letters ☐ Parcel delivery ☐ Parcel collection ☐ Storage of items
☐ Others _____________________________ (specify)

18. Have you ever heard about the term ‘smart locker’?
   ☐ Yes ☐ No (skip to section 2)

19. Which of these features does a smart locker possess?

☐ Storage compartment ☐ Padlock and key ☐ Electronic keypad ☐ Camera
☐ Display screen ☐ Audio communication system

Section 2: **FEASIBILITY and ACCEPTABILITY OF SMART LOCKERS**

A smart locker is as an automated delivery machine that has compartments used for storage of items. It as the following features; Electronic keypad, visual display screen, security camera and audio communication system. Patient medication can be placed within a locker at a convenient location for pick up. A pin code is then sent to their mobile device, which can be used to access the locker.

The picture below is a smart locker

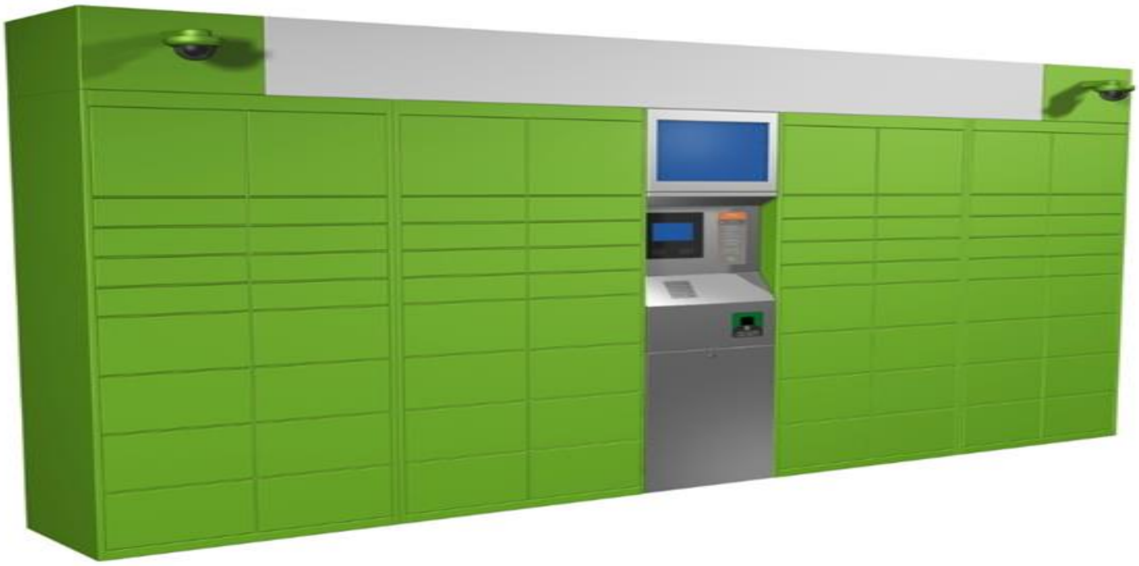

20. How likely are you to recommend this method for dispensing medication to patients living with chronic illness?

☐ Very likely ☐ Somewhat likely ☐ Not likely

21. In your opinion, how likely are your patients to accept this method for collecting their drugs?

☐ Very likely ☐ Somewhat likely ☐ Not likely

22. Which of these patients might benefit from using the smart lockers to collect their medication?

☐ Patients with HIV ☐ Patients with cancer ☐ Patients with diabetes
☐ Patients with Hypertension ☐ Patients with Tuberculosis
☐ Others ______________________________ (specify)

23. For which of these age groups can the smart lockers be used in dispensing medication?

☐ 1-12 years ☐ 13 - 17 years ☐ 18 – 40 years ☐ 41- 60 years ☐ Above 60 years

24. How might patient orientation be conducted on the use of the smart lockers?

☐ Through audio-visuals ☐ Through printed material
☐ One on one counselling by healthcare provider ☐ Other _______________________ (specify)

25. Who might be most suited to load the drugs into the smart lockers?

☐ Pharmacist or other pharmacy staff ☐ Nurse
☐ Monitoring and evaluation (M&E staff) ☐ HIV case manager
☐ Specific staff employed for this sole purpose ☐ Other ______________________ (specify)

26. What would be the optimum location for placing the smart lockers for access to patients?

☐ Hospital or clinic ☐ Community pharmacy ☐ Religious institution
☐ Police station ☐ Bus stop ☐ Other ______________________________ (specify)

27. How best might patients receive notification of drug dispensing?

☐ Email ☐ Text message ☐ Phone call ☐ Other __________________________ (specify)

28. What operating time would you recommend for patients to access the smart lockers?

☐ Morning (6am-12pm) ☐ Afternoon (12-4pm) ☐ Evening (4pm-10pm) ☐ 24 hour access

Indicate to what extent you agree with these statements about the use of smart lockers for dispensing chronic disease medication<br>**SA** – Strongly agree **A** - Agree **N** – Neither agree nor disagree **D** - Disagree **SD** – Strongly disagree					
	**SA**	**A**	**N**	**D**	**SD**
29. Lead to poor drug adherence by patients					
30. Lead to poor clinical adherence by patients					
31. Make it harder for healthcare providers to track patients health status					
32. Accrue high maintenance costs					
33. Lead to increased stigmatization and discrimination of patients living with chronic diseases					
34. Reduce the workload of healthcare workers					
35. Reduce the strain on healthfacility resources					
36. Contribute to decongestion of health facilities					
37. Lead to reduced patient waiting time					

Annex 2: Patient Survey Questionnaire

PATIENT SURVEY ON THE FEASIBILITY OF SMART LOCKERS FOR DISPENSING CHRONIC DISEASE MEDICATION IN NIGERIA

This survey contains questions that are designed to gather your perception on: the barriers to providing accessing care and treatment for chronic illness, the use of smart lockers for collecting prescribed chronic disease medication, challenges and benefits of using the smart lockers for dispensing chronic disease medication. The goal of the survey is to assess the feasibility and acceptability of using smart lockers for dispensing chronic disease medication in Nigeria. Your participation in this study is voluntary. No personal information will be collected from you. Your response will be treated confidentially. If you are willing and able to participate in this study, kindly tick the box below.

☐ I agree to participate in this study

1. Gender

   Male ☐ Female ☐

2. Age (as at last birthday)

   ☐ Less than 18 years ☐ 18- 35 years ☐ 36-60 years ☐ Above 60 years

3. State of residence ___________________________ LGA of residence _________________________

4. Marital status

   ☐ Single ☐ Married ☐ Separated ☐ Divorced ☐ Widowed

5. What is your highest educational qualification?

   ☐ No formal education ☐ First school leaving certificate ☐ WAEC/GCE/NECO

   ☐ Bachelor's Degree/HND ☐ Masters/MBBS ☐ PhD/DrPH/DA/DBA

   Others____ (specify)

6. Employment Status

   ☐ Employed ☐ Not employed

7. Which of the following categories do you belong to?

   ☐ I am currently living with a long term/ chronic illness ☐ I am a caregiver to one or more persons living with a long term/ chronic illness

8. Which chronic illness are you currently living with?

   ☐ Diabetes ☐ Hypertension ☐ Stroke ☐ HIV

   ☐ Cancer ☐ Tuberculosis ☐ Other ______________________________ (specify)

9. How long have you or the person(s) you provide care for been living with the illness?

   ☐ Less than 6 months ☐ 6 months-1 year ☐ 1 – 5 years ☐ 6 – 10 years ☐ More than 10 years

10. Are you currently undergoing treatment or management of the chronic illness?

    ☐ Yes ☐ No

11. What kind of treatment are you currently undertaking for the chronic illness? (select all that apply)

    ☐ Herbal therapy ☐ Drugs ☐ Physical therapy ☐ Behavioural therapy

    ☐ Other ____ (specify)

12. How often do you refill your drug prescription?

☐ Monthly ☐ Bi-monthly ☐ Every three months

☐ Every six months ☐ Whenever I run out of medication

13. From where do you obtain the drugs?

☐ Local pharmacy ☐ Hospital or clinic where I am registered
☐ Community-based delivery by healthcare provider ☐ Other ______________________ (specify)

14. On an average, what is your waiting time when you go for refill?

☐ Less than 10 minutes ☐ 10 – 30 minutes ☐ 30 minutes – 1hour patients
☐ More than 1 hour I ☐ don't know

15. What is the average cost of transportation to refill your drugs?

☐ 100 -200 Naira ☐ 200 – 500 Naira ☐ 500 – 1000 Naira ☐ Above 1000 Naira
☐ I don't know

16. Which of these is a barrier accessing treatment for your illness? (select all that apply)

☐ Cost of medication ☐ Distance to access care ☐ Cost of transportation to the hospital
☐ Drugs for my condition are rare ☐ Stockout of drugs ☐ Long waiting periods

17. In which of these instances have you used a locker for storage of items? (select all that apply)

☐ Banking hall ☐ Post office box ☐ Gym locker ☐ Spa or beauty salon ☐ Supermarket
☐ I have not used a locker before (skip to Section 2)
☐ Other ________________________________ (specify)

18. Which of these features does the locker you have used possess?

☐ Storage compartment ☐ Padlock and key ☐ Electronic keypad ☐ Camera
☐ Display screen ☐ Audio communication system

19. Have you made use of a storage locker that has these features: Storage compartment, Electronic keypad, Camera, Audio communication system

☐ Yes ☐ No

20. How have you made use of the locker?

☐ Posting of letters ☐ Parcel delivery ☐ Parcel collection ☐ Storage of items
☐ Others ____________________________ (specify)

21. Have you ever heard about the term 'smart locker'?

☐ Yes ☐ No (skip to section 2)

22. Which of these features does a smart locker possess?

☐ Storage compartment ☐ Padlock and key ☐ Electronic keypad ☐ Camera
☐ Display screen ☐ Audio communication system

Section 2: **FEASIBILITY and ACCEPTABILITY OF SMART LOCKERS**

A smart locker is as an automated delivery machine that has compartments used for storage of items. It as the following features; Electronic keypad, visual display screen, security camera and audio communication system. Patient medication can be placed within a locker at a convenient location for pick up. A pin code is then sent to their mobile device, which can be used to access the locker.

The picture below is a smart locker

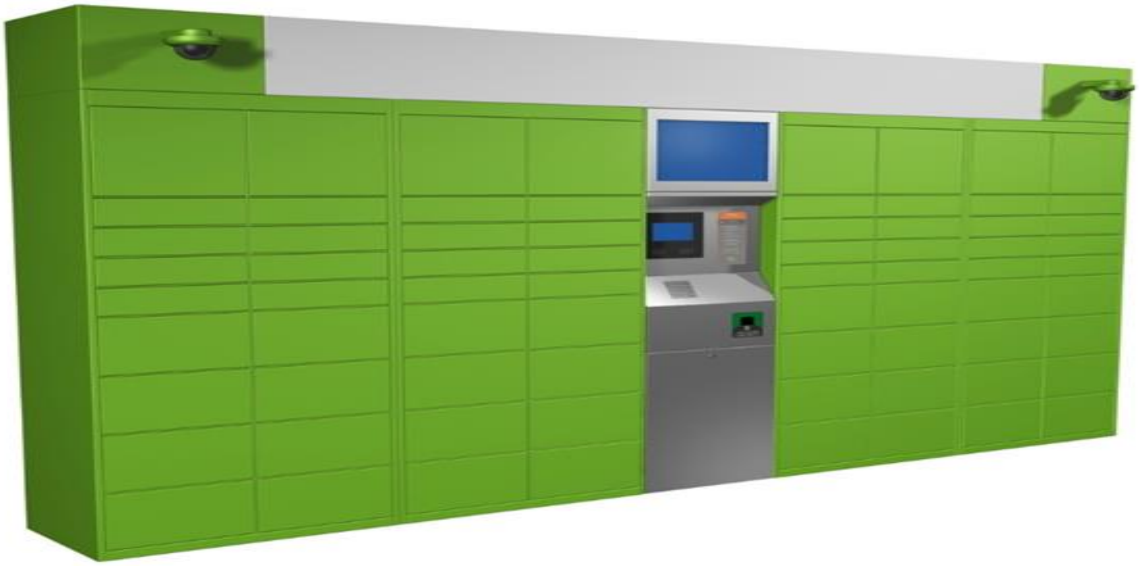

23. How likely are you to accept this method for collecting your drug refills?

☐ Very likely ☐ Somewhat likely ☐ Not likely

24. Which of these patients might benefit from using the smart lockers to collect their medication?

☐ Patients with HIV ☐ Patients with cancer ☐ Patients with diabetes
☐ Patients with Hypertension ☐ Patients with Tuberculosis ☐ I don't know
☐ Others ______________________________ (specify)

25. For which of these age groups can the smart lockers be used in dispensing medication?

☐ 1-12 years ☐ 13 - 17 years ☐ 18 – 40 years ☐ 41- 60 years ☐ Above 60 years
☐ I don't know

26. Which of these would best be able to use the smart lockers?

☐ Children with chronic illness ☐ Adults with chronic illness
☐ Pregnant women with chronic illness ☐ Elderly persons with chronic illness
☐ I don't know

27. How might patient orientation be conducted on the use of the smart lockers?

☐ Through audio-visuals ☐ Through printed material
☐ One on one counselling by healthcare provider ☐ Other _______________________ (specify)

28. What would be the optimum location for placing the smart lockers for easy access to patients?

☐ Hospital or clinic ☐ Community pharmacy ☐ Religious institution
☐ Police station ☐ Bus stop ☐ Others ______________________________ (specify)

29. How would you like to receive notifications to pick up your drugs after they are delivered to the locker?

☐ Email ☐ Text message ☐ Phone call ☐ Others _________________________ (specify)

30. At what time during the day would you prefer to use the lockers to pick up your drugs?

☐ Morning (6am-12pm) ☐ Afternoon (12-4pm) ☐ Evening (4pm-10pm) ☐ 24 hour access

Indicate to what extent you agree with these statements about the use of smart lockers for dispensing chronic disease medication<br>**SA** – Strongly agree **A -** Agree **N** – Neither agree nor disagree **D** - Disagree **SD** – Strongly disagree					
	**SA**	**A**	**N**	**D**	**SD**
31. Reduce transportation cost					
32. Reduce hospital waiting time					
33. Improve adherence to treatment					
34. Accrue high maintenance costs					

35. Which of these might be a challenge with the use of the smart locker for collecting your medication?

☐ No mobile phone to receive notification when the drugs are delivered

☐ Poor mobile network ☐ Privacy is not guaranteed

☐ Stigmatization and discrimination ☐ Disconnect from my healthcare provider

36. Would you be willing to pay a subscription fee to use the smart lockers for collecting your drugs?

☐ Yes ☐ No ☐ Depending on the cost

37. How much would you be willing to pay to use the smart lockers for collecting your drugs?

☐ 100 – 200 Naira ☐ 200 – 500 Naira ☐ 500 – 1000 Naira ☐ Above 1000 Naira
