# Supplementary material for: Acceptability of smart locker technology for dispensing chronic disease medication among patients and healthcare providers in Nigeria
Source: PLoS One. 2024 Mar 7;19(3):e0294936. doi: 10.1371/journal.pone.0294936 (PMC10919599; doi:10.1371/journal.pone.0294936)
Supplement: S4 Appendix — (PDF) [file pone.0294936.s004.pdf]

GEORGETOWN UNIVERSITY

EXEMPTION

January 4, 2022

Ibrahim Gobir

ibg7@georgetown.edu

Dear Ibrahim Gobir:

On 1/4/2022, the IRB reviewed the following submission:

Type of Review:	STUDY
Title:	Feasibility and acceptability of “smart lockers” for dispensing chronic disease medication in Nigeria
Investigator:	Ibrahim Gobir
IRB ID:	STUDY00004560
Review Type:	Non-Committee
Review Level:	Exempt
Review Category:	(2)(ii) Tests, surveys, interviews, or observation (low risk)
Funding:	Name: GUMC Dean of Research
IND, IDE, or HDE:	None
Documents Reviewed:	CITI Cert_Research Inv_Ayodotun Olutola.pdf<br>Group 2.Certificate_Gobir BI.pdf<br>Group 2.Certificate_Niyang MP.pdf<br>GU-HRP-502 - Georgetown ICF_Smart Locker_IBG_12142021.docx<br>Healthcare Provider Questionaire<br>Patient Questionaire<br>Study Protocol_Smart Locker feasibility survey in Nigeria_IBG_12142021.docx

The IRB has granted exemption to the submission. You can begin research activities. **The exemption is valid beginning 1/4/2022.** Any modifications to the protocol and other supporting documents must be reviewed by the IRB prior to implementation.

In conducting this protocol, you are required to follow the requirements listed in the Investigator Manual (HRP-103), which can be found by navigating to the IRB Library within the IRB system.

Sincerely, Cindi Charles
