# Supplementary material for: Acceptability of smart locker technology for dispensing chronic disease medication among patients and healthcare providers in Nigeria
Source: PLoS One. 2024 Mar 7;19(3):e0294936. doi: 10.1371/journal.pone.0294936 (PMC10919599; doi:10.1371/journal.pone.0294936)
Supplement: Attachment — Submitted filename: comments for Feasibility and Acceptability of Smart lockers.docx [file pone.0294936.s005.docx]

Title of Article:	Feasibility and Acceptability of Smart lockers Technology for Dispensing Chronic Disease Medication in Nigeria

Document type:	Manuscript

Corresponding author:	Ibrahim Bola Gobir, MD, MPH, Georgetown University Medical Center

Proposed Journal:	PLOS

Reviewed by:	Mukhtar Liman AHMAD

Date reviewed:	August 26, 2023

Recommendation:	Manuscript needs to be revised to effect the comments

Minor Comments

1. Line 23 -24: Study was conducted in 6 states but only 5 states were mentioned. Also 5 states was mentioned on Line 67. Authors should rectify the numbers of states used.
2. The following references cited were not found on the reference list, authors should consider adding them to the list of references:
   a. Line 80: According to Faronbi, Ademuyiwa and Olaogun (2020) …..
   b. Line 87: According to Adebayo, Labiran, Emerenini and Omoruyi (2016), …
3. The citation style was mixed in the text. Please ensure that the reference style adheres PLOS ONE guidelines.
4. Line 198 – 201: Review the text and consider rephrasing/revising sentences to improve reader clarity.

Major Comments

1. The study was limited to only 5 or 6 states, but the sample size was computed based on some national estimated parameters (Nigerian population, percentage of chronic diseases and estimated number of HCWs). I saw some elements of over sampling of the participants which would have likely affected the entire statistical analyses and render the results void.
2. What is the rational of using 4% error margin when computing sample size for PATIENTS and 5% error margin when computing sample size for HCWs?
