# Supplementary material for: Acceptability of smart locker technology for dispensing chronic disease medication among patients and healthcare providers in Nigeria
Source: PLoS One. 2024 Mar 7;19(3):e0294936. doi: 10.1371/journal.pone.0294936 (PMC10919599; doi:10.1371/journal.pone.0294936)
Supplement: Attachment — Submitted filename: Response Letter to Reviewers Manuscript 1_SLS_FINAL.docx [file pone.0294936.s006.docx]

Dear Drs Ibrahim Jahun and Dr Mukhtar Liman Ahmed,

Thank you for providing valuable feedback and giving us the opportunity to resubmit a revised draft of our manuscript, now titled “Acceptability of Smart Lockers Technology for Dispensing Chronic Disease Medication among Patients and Health Care Providers in Nigeria.”

We appreciate the efforts put into the details, including the time and resources dedicated to providing these valuable comments and reviews. We have incorporated the changes to reflect most of the suggestions provided and to put into context some of the comments in the paper.

Reviewer 1(Mukhtar Liman Ahmed): Thank you for your comment on number of states, it has been clarified and addressed: 5 states were included in the study according to Appendix 1 and the result.

Reviewer 1(Mukhtar Liman Ahmed): It is deeply appreciated that you pointed out the missed references, we have now included these references in the reference list using the PLOS ONE reference style.

Reviewer 1(Mukhtar Liman Ahmed): Thank you for pointing the mixed references, they have been cited properly and the reference style adjusted accordingly and consistently.

Reviewer 1(Mukhtar Liman Ahmed): Thank you for this feedback, the sentence has been revised and rephrased to be adjusted in line with the result of the study to improve clarity.

Reviewer 1(Mukhtar Liman Ahmed): Thank you for pointing this out the number of on the mixed number of states and the sample sizes based on the national estimated parameters. The study was conducted in 5 states, and we have corrected this. We agree that using national estimates may not be ideal. However, we used these estimates because we believe national estimates may be appropriate for a multi-state study than using single study estimates. We oversampled to account for the inherent nature of poor response rates that have been traditionally reported for online surveys.

Reviewer 1(Mukhtar Liman Ahmed): Thank you for your keen observation on the rational for the error margins. We varied the error margins to adjust for differences in expected precision based on the calculated sample sizes of the two study populations.

Reviewer 2(Ibrahim Jahun, Academic editor): Thank you for your comment on financial disclosure. The study was funded by Georgetown University Medical Center, Dean of Research. The funder did not play any role in the research. This has been addressed in the cover letter and the section allotted for funding information in the submission form.

Reviewer 2(Ibrahim Jahun, Academic editor): Thank you for your comment on the criteria for facility selection. The facilities were identified and randomly selected via implementing partners supporting clinical care.

Reviewer 2(Ibrahim Jahun, Academic editor): Thank you for bringing our attention to the missing references. They have been included.

Reviewer 2(Ibrahim Jahun, Academic editor): This comment is well received on the inclusion criteria for our study group. The inclusion criteria for the study were individuals who are either or receiving care from facilities listed in Appendix 1”. We collected information from healthcare providers and patients. For a few of the patients who were not able to complete the survey by themselves, this was completed by their relative on their behalf. The relatives were not part of the study population and we have updated that.

Reviewer 2(Ibrahim Jahun, Academic editor): This comment on patients/ care providers is appreciated and noted. Our document reflects “patients” going forward.

Reviewer 2(Ibrahim Jahun, Academic editor): Thank you for your question on whether the study was conducted in HIV clinics. The study was conducted among patients with chronic illnesses and Health Care Workers which were identified through ART programs’ implementing partners (IP). However, for this study, we included patients from other general and disease-specific clinics within the same hospitals. We did not specifically set out to screen for HIV in other clinics. However, we agree that there is a possibility of comorbidity with HIV in other non-HIV clinics that patients may be unaware of.

Reviewer 2(Ibrahim Jahun, Academic editor): This is kindly noted and adjusted. Our document reflects “patients” going forward.

Reviewer 2(Ibrahim Jahun, Academic editor): This comment is kindly noted. This adjustment has been made in this section of the result to include “in 5 states in Nigeria.”

Reviewer 2(Ibrahim Jahun, Academic editor): We have included the proportions and 95% CIs of the proportions for acceptability across different chronic diseases.

Reviewer 2(Ibrahim Jahun, Academic editor): Thank you for your observation on the lack of naira equivalent to USD. Yes, willingness to pay gives further justification for the acceptability of smart lockers. The Naira equivalent to USD at the time of the study has been included.

Reviewer 2(Ibrahim Jahun, Academic editor): thank you for sharing your observation on the hanging subheading “Barriers to accessing and providing chronic disease care among patients and healthcare workers in Nigeria”. This is kindly noted. This section has been addressed in the objectives, results and the discussion section.

Reviewer 2(Ibrahim Jahun, Academic editor): We agree with the reviewer that we did not define feasibility in this paper. We did not set out to assess feasibility on its own but indirectly. Our aim was to directly measure acceptability of and willingness-to-pay for smart locker and indirectly use them as indicators of feasibility of this intervention in Nigeria.

Reviewer 2(Ibrahim Jahun, Academic editor): The comment on defining feasibility is well received. We have now updated the title to reflect this: “Acceptability of smart locker technology for chronic disease medication among patients and healthcare providers in Nigeria.”

Reviewer 2(Ibrahim Jahun, Academic editor): Thank you for your comment on description of questionnaire, this is noted and has been addressed and a description of the questionnaire has been included in the methods section.

Additional Clarifications

1. The PLOS ONE File naming format was used as requested.
2. The PLOS ONE questionnaire on Inclusivity in global research is not required for this paper as this applies to researchers who travelled to a different country to conduct research.
3. The full ethics statement of our study has been included in the methods section, as requested.
4. The tables referred to (6 & 8) on pages (10 &11) have been merged into tables (4 & 5) based on relevant information required for the tables.
5. All in-text citations have been updated to match accordingly.

We look forward to hearing from you in due time regarding our submission and to responding to any further questions and comments you may have.

Sincerely,

Ibrahim Bola Gobir.
